# A Critical Analysis of the Conventionally Employed Creep Lifing Methods

**DOI:** 10.3390/ma7053371

**Published:** 2014-04-29

**Authors:** Zakaria Abdallah, Veronica Gray, Mark Whittaker, Karen Perkins

**Affiliations:** Institute of Structural Materials, College of Engineering, Swansea University, Swansea SA2 8PP, UK; E-Mails: v.a.gray@swansea.ac.uk (V.G.); m.t.whittaker@swansea.ac.uk (M.W.); k.m.perkins@swansea.ac.uk (K.P.)

**Keywords:** creep, creep lifing methods, long-term creep behaviour, stress rupture, the Wilshire equations

## Abstract

The deformation of structural alloys presents problems for power plants and aerospace applications due to the demand for elevated temperatures for higher efficiencies and reductions in greenhouse gas emissions. The materials used in such applications experience harsh environments which may lead to deformation and failure of critical components. To avoid such catastrophic failures and also increase efficiency, future designs must utilise novel/improved alloy systems with enhanced temperature capability. In recognising this issue, a detailed understanding of creep is essential for the success of these designs by ensuring components do not experience excessive deformation which may ultimately lead to failure. To achieve this, a variety of parametric methods have been developed to quantify creep and creep fracture in high temperature applications. This study reviews a number of well-known traditionally employed creep lifing methods with some more recent approaches also included. The first section of this paper focuses on predicting the long-term creep rupture properties which is an area of interest for the power generation sector. The second section looks at pre-defined strains and the re-production of full creep curves based on available data which is pertinent to the aerospace industry where components are replaced before failure.

## 1. Introduction

There are many applications where materials are required to operate for long periods in severe environments of high stresses and temperatures without failure. Examples of this are the power generation industry and the gas turbine sector where components are experiencing higher temperature environments as designers seek to increase thermal efficiency. Because of these harsh engineering environments, both areas are dependent on appropriate methods of creep lifing even though the specific requirements of each industry differ significantly. In the power generation sector, a significant part of the industry entails static parts experiencing pressure loads where creep is often the most critical damage mechanism. These plants are designed to operate for periods in excess of 100,000 h and often to 250,000 h, and, with vast amounts of material being required, it is clear there is an opportunity for significant cost savings through the employment of new alloys. However, working over such long timeframes means that it is not feasible to conduct 100,000 h creep tests on new materials in order to establish safe operating stresses for the life of the plant. Therefore, appropriate extrapolation methods are required in order to derive crucial design stress limitations from short term creep tests.

Many approaches to predicting creep have been proposed in an effort to infer long-term deformation behaviour. As such, parametric creep methods play a key role in design as they are essential in predicting the specific life of high temperature components [1,2]. Each of these approaches is a technique where short-term creep-rupture data can be extrapolated to longer-term scenarios using a time-temperature parameter. This concept is based on the assumption that all creep-rupture data for a given material can be combined to produce a single “master curve” where stress is mapped against a parameter that combines the effect of time and temperature. This can then be used to extrapolate to longer time frames such as those experienced in the power generation sector.

Contrastingly, for a gas turbine engines there are different design requirements due to the number of rotating parts which are more likely to fail from fatigue rather than creep. However, fatigue damage models rely on input stresses, which are dependent on stress relaxation, governed by creep deformation. Accurate creep models which describe creep strain evolution, particularly for relatively low creep strains, are therefore extremely desirable. Clearly, the goal of both industries is an all encompassing model capable of each of these requirements whilst being transferable across materials and providing confidence through supportive observations from experimental data. This is a goal which thus far has eluded engineers. Indeed, as recently as 2010 the extrapolation of creep data was rated as the most important challenge in the UK Energy Materials Strategic Research [3].

This paper critically reviews some of the most commonly used creep lifing methods available. It is structured with two themes; models capable of creep life extrapolation, and, models able to re-produce full creep curves based on limited creep data. Creep life extrapolation methods are evaluated using the National Institute of Materials Science (NIMS) creep data of a well-understood power generation alloy, Grade 22 Steel (2.25Cr-1Mo) [4]. Reproduction of creep curves from limited data is undertaken for the aerospace alloy Titanium 834. It should be noted that this paper is not an exhaustive review of all possible creep models but rather an overview and evaluation of the most commonly employed creep models. Previous studies have undertaken more thorough reviews of traditional methodologies providing guidance on acceptability criteria [5,6]. These criteria assisted in the assessment of methodologies explored in this paper prior to application of a mean squared error-based approach.

## 2. Approaches to Creep Life Predictions

Generally, the traditional approach to creep lifing has been based on a power law relationship between temperature, *T* and stress, σ. Arrhenius [1] relates these parameters to the creep rate through:

$${έ}_{S}\;\alpha \text{ exp}(-Q_{c}/RT)$$ (1)

and by Norton [1]:

$${έ}_{S}\;\alpha \;\sigma^{n}$$ (2)

where έ_s_ is the secondary or steady-state creep rate; *Q*_c_ is the activation energy for creep; n is the stress exponent and *R* is the gas constant. Combining these two relationships yields the basic power law Equation (1):

$${έ}_{S}=A\cdot \sigma^{n}\cdot \text{exp}(-Q_{c}\text{/}RT)$$ (3)

where A is a proportionality constant. The initial assumption when this law was introduced was that *Q*_c_ and n are constants. However, after further research, it was observed that these “constants” varied according to the imposed creep conditions and mechanisms involved in different stress and temperature regimes [7]. With pure metals, a decrease from *n* ≅ 4 to *n* ≅ 1 with decreasing stress, has been widely attributed to a change from diffusion-controlled dislocation processes to diffusional creep mechanisms not involving dislocation movement. However, at high stresses strengthened alloys with fine precipitate or insoluble particles can produce values of *n* >> 4 where the value for *Q*_c_ easily exceeds that of the activation energy for lattice diffusion, *Q*_SD_ [8]. No generally accepted interpretation has been agreed upon to account for these anomalously large n and *Q_c_* values.

### 2.1. Traditional Power Law Creep Models

A wide variety of parametric approaches to creep lifing based on the above power law equation, Equation (3), have been developed over the past 60 years with varying degrees of success. A number of the most widely used power law approaches are explored in this section.

#### 2.1.1. The Larson-Miller Approach

This parametric method is one of the most widely employed techniques for stress-rupture behaviour prediction in metals. It was originally derived from the basic power law equation, Equation (3), by assuming a constant stress over a variable temperature range. The Larson-Miller approach is given by [9]:

$$P_{\text{LM}}=f(\sigma )=T\cdot (C_{\text{LM}}+\text{log}t_{f})$$ (4)

where *C*_LM_ and *P*_LM_ are the Larson-Miller constant and parameter, respectively.

Larson and Miller expanded their original proposal suggesting that the value of *C*_LM_ to be taken as 20 for metallic materials [9,10]. This presented a problem because if the value of *C*_LM_ is universally 20 for all metallic materials, then this implies that for the same testing conditions the time to fracture is the same for all metallic materials. In addition, this suggestion means that for a given material, once a set of stress-rupture curves at different temperatures are established, then over the same temperature range these curves would be valid for any other material provided the stress scale is altered [11]. However, it was subsequently found that the value *C*_LM_ varies from one alloy to another and is influenced by factors such as cold-working, thermo-mechanical processing, phase transitions and/or other structural modifications [10]. To address this complexity, *C*_LM_ is a “fitted constant” acquired purely from the data with no physically meaningful description.

Although the Larson-Miller approach is widely used to model creep, it does have inherent limitations. Even though *C*_LM_ is given as a constant, it is known from observation that it is not constant for all conditions [7]. Furthermore, the parameters and constants described here have yet to be linked to any physical phenomena. Therefore, what the constants represent and how to predict them remains unclear, highlighting two common issues for many creep models. The first issue is that these empirical equations employ constants which upon investigation are often no longer constant over the conditions considered. The second is that the parameters used in the equations cannot be linked to any physical phenomena and hence, it is unclear what they represent. This means that it is difficult to expand such models to be able to extrapolate more widely and include factors such as cold-working, thermo-mechanical processing, phase transitions and/or other structural modifications known to change creep properties.

#### 2.1.2. The Manson-Haferd Approach

Manson and Haferd [12] developed a linear time-temperature relationship for creep and stress-rupture data. This methodology was introduced in order to address the inaccuracy of the Larson-Miller approach resulting from the suggestion of a fixed *C*_LM_ for metallic materials [12,13]. The Manson-Haferd approach, like the Larson-Miller, assumes steady-state creep is dominated by power law behavior. However, the innovation of the Manson-Haferd approach assumes that the logarithm of the time varies linearly with temperature at a constant initial stress, according to [12,14]:

$$P_{\text{MH}}=f(\sigma )=(\text{log}t-\text{log}t_{a})/(T-T_{a})$$ (5)

where *P*_MH_, *t*_a_ and *T*_a_ are the Manson-Haferd parameter, time and temperature constants, respectively. The variable *t* refers either to the time to fracture, *t*_f_, or, to a pre-defined strain level, *t*_ε_, and T is the absolute temperature of the creep test. Mapping iso-stress data as a function of time and temperature, *i.e*., *f*(σ) = *f*(*t*, *T*) at σ = constant, *t*_a_ and *T*_a_ are obtained from the intercepts of a linear fit, *i.e*., at (*t*, *T*) → (*t*_a_, 0), (0, *T*_a_). This means that the Manson-Haferd parameter, *P*_MH_, is composed of two constants unlike the Larson-Miller parameter, *P*_LM_, which involves only one. The Manson-Haferd parameter, *P*_MH_, can therefore be derived graphically from the point of intersection of the extrapolated iso-stress lines of log*t*_f_ *vs*. T. Moreover, plotting *P*_MH_ *vs*. σ, forces all creep data to collapse onto a single “master curve” providing an equation that relates the stress to time and temperature [12].

The perceived advantage of this method over the Larson-Miller’s approach is that the parameter, P_MH_, is composed of two constants relating time to temperature, thus, possibly giving this model better sensitivity to the time-temperature relationship. Once again, the physical meaning of *t*_a_ and *T*_a_ is not known and as such what they represent is unclear.

#### 2.1.3. The Orr-Sherby-Dorn Approach

The Orr-Sherby-Dorn (OSD) [15] approach involves a time-temperature constant, *C*_OSD_, based on the linear relationship of log t *vs*. 1/T. In the OSD approach, the initial premise of Larson and Miller has been modified such that the constant *C*_LM_ becomes a function of stress, and *P*_LM_ becomes a constant [13,14]. Based on these assumptions, the Larson-Miller relation, Equation (4), can be re-arranged to yield the Orr-Sherby-Dorn equation [15]:

$$P_{\text{OSD}}=f(\sigma )=\text{log}t_{f}-C_{\text{OSD}}/T$$ (6)

where *P*_OSD_ and *C*_OSD_ are the Orr-Sherby-Dorn parameter and constant, respectively. This method offers a different perspective on the relationship between parameters and constants which leads to it producing smooth creep curves rather than sigmoidal ones. This distinction in the curve shape is one reason the OSD method may produce better creep rupture predictions than other methods.

Once again, this approach suffers from its empirical nature. Similar to the Larson-Miller and Manson-Haferd approaches, the OSD utilises a constant, *C*_OSD_, based on *Q*_c_ which is usually obtained from sparse data [15]. The effect of this is that any variation in *Q*_c_ will consequently ensure that the superimposed parametric plots will be non-linear [16]. Indeed, there is evidence that in some cases, creep activation energy appears to systematically increase through the primary creep region [17]. The variation in the value of *C*_OSD_ has been observed experimentally by Murray and Truman [18]. Given that *C*_OSD_ is the gradient of logt_f_ *vs*. 1/T, it was proposed by OSD that the adjacent logσ *vs*. log*t*_f_ curves will be equidistant from each other along the time scale [11]. In principle, this approach can be used by obtaining *C*_OSD_ from a single plot, but in reality, *C*_OSD_ is usually an average value obtained from a number of log*t*_f_ *vs*. 1/*T* values.

#### 2.1.4. The Manson-Succop Approach

The Manson-Succop approach [19] describes the iso-stress values to be proportional to log*t*_f_ and *T*. This differs from OSD method which relates the iso-stress data to log*t*_f_ and 1/*T*. The Manson-Succop parameter and constant, *P*_MS_ and *C*_MS_, are related by [19]:

$$P_{\text{MS}}=f(\sigma )=\text{log}t_{f}+C_{\text{MS}}\cdot T$$ (7)

This approach produces similarly shaped curves to the OSD approach, however, differs in its method of describing the time-temperature relationship. This method was reviewed by Zharkova and Botvina [20] who confirmed that during long-term creep tests, fracture mechanisms changed according to the applied stress and the loading time. Although the mechanism changed, it was concluded that the derived constant did not change for the Manson-Succop, Larson-Miller or Manson-Haferd approaches. In the opinion of these authors [20], this claim has not been definitively adopted as there is evidence to both support and counter the idea that the constants in these approaches are independent of the creep mechanism.

#### 2.1.5. The Goldhoff-Sherby Approach

This approach is similar to the Manson-Haferd method with the difference being that the constructed iso-stress lines will converge to a point (1/*T*_a_, *t*_a_) such that [21]:

$$P_{\text{GS}}=f(\sigma )=(\text{log}t-\text{log}t_{a})/(1/T-1/T_{a})$$ (8)

where *t_a_* and *T*_a_ are the time and temperature constants, respectively. The mathematical procedures are similar to those applied using the Manson-Haferd approach, however, log*t*_f_ is plotted against 1/*T* at constant stresses with the slope being the value of the Goldhoff-Sherby parameter, *P*_GS_. An average value for log *t*_a_ and 1/*T*_a_ should then be chosen in order to construct the stress *vs*. time to fracture curves. This model contains the potential time-temperature sensitivity of the Manson-Haferd but offers a slightly different curve shape.

#### 2.1.6. The Soviet Prediction Approach

This method can be described by two models; Soviet models (1) and (2), given by [22,23]:

Soviet Model (1):logt=a+blogT+clogσ+d/T+f⋅σ/T (9)

Soviet Model (2):logt=a+blogT+clog(σ/T)+d⋅σ/T+f/T (10)

where *a*, *b*, *c*, *d* and *f* are constants to be determined from fitting the equations to data. This method uses a large number of constants which allows for greater sensitivity, but the constants are not easily separable from the variables *T* and σ, meaning that multiple constants need to be fitted simultaneously. This might result in more than one possible set of values for *a*, *b*, *c*, *d* and *f*. From the experience of the authors of this paper, fitting constants can lead to unphysical curves and this should be noted when employing this method. A means of fitting these multiple constants simultaneously is provided by the software PD6605 which iterates the numerical coefficients of each equation, first to optimise their values and then to determine which has the greatest likelihood of providing the most accurate results [24]. Evans [22] expressed that Soviet Model (1) was highly effective in modeling the known rupture times, but was totally inadequate for predicting beyond the range of input data. However, this inability to generalise or the tendency to overfit the interpolative data set, is a characteristic of all parametric techniques [22].

#### 2.1.7. The Minimum Commitment Approach

This method was proposed by Manson and Ensign [25,26] in an effort to give larger flexibility to parametric analysis of creep data. In addition, it was proposed as a way to unite conflicting approaches into a single equation with sufficient generality. The Minimum Commitment approach is given by [25,26]:

$$\text{log}t=a+b\text{log}\sigma +c\cdot \sigma +d\cdot \sigma^{2}+f\cdot T+g/T$$ (11)

In the above equation there are six constants that need to be determined via regression analysis. Similar to the Soviet approach, the large number of constants coupled to variables introduces analysis issues when performing any fitting process but gives greater detail and sensitivity to the time-temperature-stress relationship. Among those who have studied this methodology was Jow-Lian Ding *et al*. [27] who found that the results of regression analyses indicated that the Minimum Commitment model fitted the creep data studied only slightly better than the Larson-Miller model regardless of the high number of constants.

### 2.2. Modern Creep Lifing Approaches

#### 2.2.1. The Hyperbolic-Tangent Approach

Developed by Rolls-Royce plc (London, UK) in the 1990s for creep strain and life predictions, this approach implements the idea that the highest stress that can be applied to a specified material at a certain creep temperature is the ultimate tensile strength, σ_TS_, of the material. The stress rupture behaviour is described by hyperbolic tangent curves over a wide range of temperatures, such that [28–30]:

$$\sigma =\sigma_{\text{TS}}/2\{1-\text{tan}h[k\cdot \text{log}(t/t_{i})]\}$$ (12)

where *k* and *t*_i_ are fitting parameters that can be obtained via regression analysis by plotting at constant temperatures, tan*h*^−1^(1 − 2(σ/σ_TS_)) *vs*. log*t*_f_. Once these values are obtained, they can then be inserted into Equation (12) to produce the stress rupture curves. The inflection point of these curves at σ = 0.5σ_TS_ provides a different shape to that of the previous methods and therefore may provide a better fit for creep data with changes in mechanism that significantly impact creep lifetime.

#### 2.2.2. The Wilshire Approach

Developed at Swansea University by Wilshire [16,31], this methodology has been applied widely for long-term creep predictions [16,31]. The innovation of this method is that the stress applied is normalised by the ultimate tensile strength, σ_TS_. The stress can also be normalised by the yield stress, σ_y_, however, the obtained data fit is generally less accurate as the value of σ_y_ is more difficult to be precisely measured than the σ_TS_ [32]. Normalisation by the σ_TS_ also provides the advantage of values of (σ/σ_TS_) being between 0 and 1, meaning that property comparisons for different metals and alloys can be significantly simplified [31].

Normalising the applied stress in the power law equation, έ_m_ = *A*·σ*^n^*·exp(−*Q*_c_/*RT*), and defining the minimum creep rate, έ_m_, as in the Monkman-Grant relationship, έ_m_ = *M*/*t*_f_, gives [16]:

$${έ}_{m}=M/t_{f}=A*\cdot {\left(\sigma \text{/}\sigma_{\text{TS}}\right)}^{n}\cdot \text{exp}(-Q_{c}\text{*/}RT)$$ (13)

where A* ≠ A and *Q*_c_* ≠ *Q*_c_. In this case, *Q*_c_* is determined from the temperature dependence of έ_m_ and/or *t*_f_ at constant (σ/σ_TS_), unlike *Q*_c_ which is normally calculated at constant σ. As it stands, this equation does not permit reliable extrapolation of the short-term measurements due to the unpredictable fall in n values as (σ/σ_TS_) decreases. However, Equation (13) reduces the scale and the number, but not the maximum duration, of experimental tests needed to be undertaken to obtain long-term strength data [16,31].

Since σ_TS_ represents the maximum stress that can be applied on a material at a specific creep temperature, the data sets can be described over the entire stress range from (σ/σ_TS_) = 1 to (σ/σ_TS_) = 0. In addition, it is evident that έ_m_ → ∞ and *t*_f_ → 0 as (σ/σ_TS_) → 1, whereas έ_m_ → 0 and t_f_ → ∞ when (σ/σ_TS_) → 0. These essential criteria are met by augmenting Equation (13) so that the stress and temperature dependences of the creep life are described by [16,31,32]:

$$\sigma \text{/}\sigma_{\text{TS}}=\text{exp}(-k_{1}{\left[t_{f}\cdot \text{exp}(-Q_{c}*/RT)\right]}^{u})$$ (14)

where the values of the coefficients *k*_1_ and *u* are fitting constants. To obtain k_1_ and u, a linear fit of log (*t*_f_·exp(*Q*_c_*/*RT*)) *vs*. log(−log(σ/σ_TS_)) is produced with gradient equal to *u*, and, intercept equal to log*k*_1_. This method, unlike the others, produces more physically realistic curves as έ_m_→ ∞ and *t*_f_ → 0 when (σ/σ_TS_) → 1, and έ_m_ → 0 and *t*_f_ → ∞ when (σ/σ_TS_) → 0. It also has only two fitting constants easily determinable from limited data that remain conditionally constant. This statement means that when plotting to obtain *k*_1_ and *u*, it has been observed that up to two linear regimes are sometimes obtained, referred to as the high and low stress regimes. This means that *k*_1_ and *u* may have different values above and below a certain normalised stress threshold thought to arise from a change in creep mechanism. Although this change in constants has been observed, *k*_1_ and *u* in their respective high and low stress regimes have so far proven to be constant.

## 3. Approaches to Reproducing Full Creep Curves

In the same manner in which stress-rupture prediction has been approached, this section considers approaches for creep strain predictions and hence the prediction of full creep curves. As stated previously, this study investigates only a small number of available methods, focusing mainly on more recently published techniques. The ECCC report published in 2003 provides a more extensive review of these and other methods [33].

### 3.1. The θ-Projection Approach

The principle of the θ-Projection approach is that creep curves under uniaxial constant stress measured over a range of stresses and temperatures can be “projected” to other stress/temperature conditions re-constructing full creep curves. The required properties can then be read off the newly constructed curves [34]. The θ-Projection concept in its most general form is the 4-θ equation [35]:

$$\varepsilon =\theta_{1}[1-\text{exp}(-\theta_{2}\cdot t)]-\theta_{3}[1-\text{exp}(\theta_{4}\cdot t)]$$ (15)

where θ_1_ and θ_3_ are “scaling” parameters that define the extent of the primary and tertiary creep with respect to strain. Whereas θ_2_ and θ_4_ are “rate” parameters characterising the curvature of the primary and tertiary creep curves [36,37]. This method has been found to provide quite poor fits to experimental creep data for small strain values [35]. Deviations from experimental creep measurements were also found particularly in the late tertiary stage by Evans and Wilshire [37] when this method was used. In response to these deviations, a modification was suggested by Evans [38] to improve the fit at very small strain values. This has been achieved by adding another two extra parameters to Equation (15) to produce 6-θ equation [38]:

$$\varepsilon =\theta_{1}[1-\text{exp}(-\theta_{2}\cdot t)]-\theta_{3}[1-\text{exp}(\theta_{4}\cdot t)]+\theta_{5}[1-\text{exp}(-\theta_{6}\cdot t)]$$ (16)

In this equation, the first two terms have the same physical meaning as in Equation (12). The additional third term describes the early primary creep behaviour that results from initial sliding relaxation across grain boundaries [36]. According to Evans [35,36,38], this modified equation provided better results when applied to creep data.

### 3.2. The Uniaxial Creep Lifing Approach

This deformation mechanism-based approach was recently developed at Rolls-Royce Canada by Wu [38] in an effort to describe the entirety of creep strain curve *vs*. time. It considers that creep deformation occurs by grain boundary sliding (GBS), dislocation glide (DXNG), dislocation climb (DXNC) and diffusion (DFN) mechanisms such that for a polycrystalline material, the total strain rate, έ, is equal to the sum of the contributions from each mechanism [39]:

$$έ={έ}_{\text{gbs}}+{έ}_{g}+{έ}_{c}$$ (17)

Re-arranging this equation and expressing the strain rate, έ, in terms of strain, ε, gives [39]:

$$\varepsilon =\varepsilon_{0}+\varepsilon_{p}[1-\text{exp}(-t/t_{\text{tr}})]+1/M[\text{exp}(M\cdot k\cdot t)-1]$$ (18)

The primary strain, ε_p_, is obtained from the linear projection of the secondary/steady-state region of the creep curve at *t =* 0. The transient time, t_tr_, is defined as the transition point from primary to secondary/steady-state creep. The slope *k* corresponds to the minimum creep rate and *M* is a factor obtained from the slope of log έ against *t*. For creep curve analysis, one needs to extract the creep curve parameters, ε_p_, *t*_tr_, *k* and *M* from actual creep strain *vs*. time curves and then apply Equation (18) to predict the creep strain accumulation (see [39]). The parameters in Equation (18) can be expressed in terms of stress and σ_TS_ such that:

$$t_{\text{tr}},k\text{ and }M=\text{f(}\sigma \text{/}\sigma_{\text{TS}}\text{)}$$ (19)

$$\varepsilon =\varepsilon_{0}+\varepsilon_{p}\cdot f(\sigma /\sigma_{\text{TS}},t)$$ (20)

The advantage of this approach is that once the parameters are expressed in terms of σ and σ_TS_ at a given temperature, Equation (18) can then be used to construct full creep curves at any specified stress for that temperature. In other words, only few tests are required at a given temperature to obtain the relationship, *i.e*., f(σ/σ_TS_), and the unknown parameters from which a database of creep curves can be generated at any given stress level outside the experimentally investigated range.

### 3.3. The Wilshire Approach

In addition to the ability to describe the stress-rupture behaviour, this technique has been extended to accurately re-construct full creep curves [40]. The time to fracture, t_f_, in Equation (14) can be replaced by the time to a pre-defined strain level, *t*_ε_, which yields [16,31,40–42]:

$$\sigma /\sigma_{\text{TS}}=\text{exp}(-k_{3}{[t_{e}\cdot \text{exp}(-Q_{c}\text{*/}RT\text{)]}}^{w}\text{)}$$ (21)

where *k*_3_ and *w*, are constants to be determined following the similar process previously described. It was found elsewhere [40] that the value of w and k_3_ used in Equation (21) is independent of stress and temperature at any selected strain level and thus they can be expressed over a range of selected strains such that:

$$w=f_{1}(\varepsilon )$$ (22)

$$k_{3}=f_{2}(\varepsilon )$$ (23)

Inserting these two expressions into Equation (21) gives:

$$\sigma /\sigma_{\text{TS}}=\text{exp(}-f_{2}(\varepsilon )\cdot [t_{\varepsilon }\cdot \text{exp}(-Q_{c}\text{*}/RT)]f_{1}\text{(}\varepsilon \text{))}$$ (24)

Re-arranging this equation yields an equation that relates the strain, ε; to stress, σ; temperature, *T*; over time, *t*, such that:

ε=f(t,σ,T) (25)

Obtaining Equation (25) means that full creep curves at various stresses and temperatures can be re-constructed.

## 4. Results

This section is divided into two parts, namely; those for the creep life prediction approaches, and, those for full creep curve representation. In the first section, the material used is Grade 22 Steel whereas for full reproduction of creep curves Titanium IMI834 creep data was employed. Grade 22 steel was selected for this review as it is a widely used material with data covering appropriate creep lives and also because it is a difficult material to effectively life, aiding in exposing limitations of the approaches assessed here. Similarly, Titanium 834 has been selected as the model material due to its wide employment in the aerospace industry, in particular the compressor of gas turbine aeroengines. The reasons for this selecting Titanium 834 are the relevance of this material to the aerospace industry and the availability of creep data.

### 4.1. Grade 22 Steel Data

**Figure 1.**
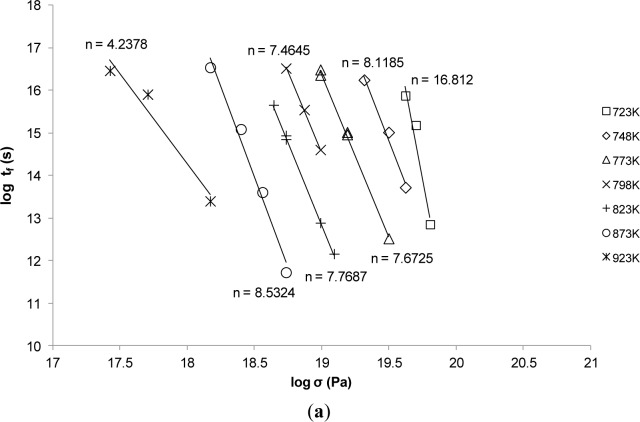

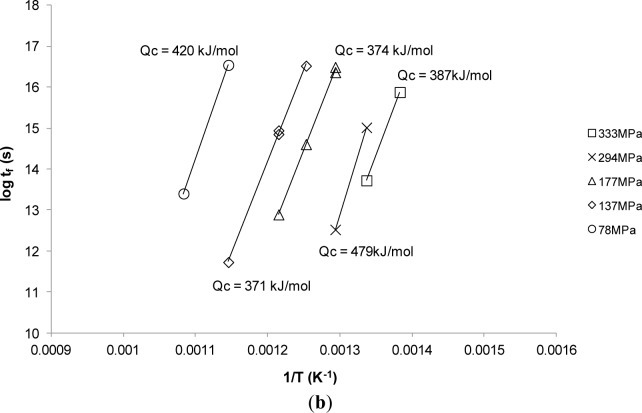
The calculation of the: (**a**) stress exponent *n*; (**b**) the activation energy *Q*_c_ at various conditions for Grade 22 Steel.

As already noted, the values of *Q*_c_ and n are important when studying creep properties. The creep data of Grade 22 Steel was obtained from the National Institute of Materials Science (NIMS). Figure 1a depicts the exponent n at various conditions whereas Figure 1b shows the value of *Q*_c_ at various conditions. From these two plots, it is evident that both n and *Q*_c_ are not constants under the investigated conditions which implies that any involvement of such constants in the parametric approaches will ensure inaccuracies in their predictions.

### 4.2. Titanium IMI834 Data

An extensive matrix of creep testing has been carried out at Swansea University using Uniaxial constant-stress creep machines performed in air according to ISO 204:2009 [43] on this material. The alloy IMI834 is widely used in the rings, compressor discs and blades for gas turbines and jet engine applications [44]. The results of these creep tests are used here for reconstructing full creep curves utilising the various approaches.

### 4.3. Creep Life Predictions (Steel 22 NIMS Creep Data)

In each creep lifing predictive approach explored, data of stress rupture lives <5000 h was used to determine the constants and parameters for each approaches and then applied to the larger complete dataset. In each of the following figures experimental values are represented as “points” and creep rupture model predictions are shown as “solid lines”.

#### 4.3.1. The Larson-Miller Approach

**Figure 2.**
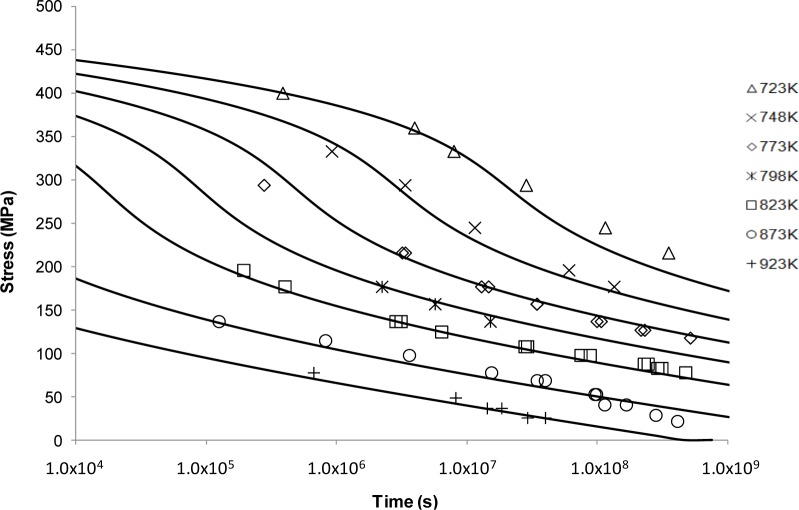
The Larson-Miller’s stress *vs*. time to fracture predictive curves using Grade 22 Steel creep data.

Using the Larson-Miller approach, log *t*_f_ *vs*. 1/*T* was plotted at constant stress with the value of *C*_LM_ assumed constant although observed otherwise [14,16,45]. Predicted curves based on <5000 h data are shown alongside the available NIMS data in Figure 2.

The fits in Figure 2 are considered reasonable for the lower temperatures. As the temperature increases, the model flattens out and no longer reflects the shape or trend of the measured data. This supports the idea that the Larson-Miller model was developed based on low temperature deformation results making extrapolation at the high temperatures inaccurate [46].

#### 4.3.2. The Manson-Haferd Approach

**Figure 3.**
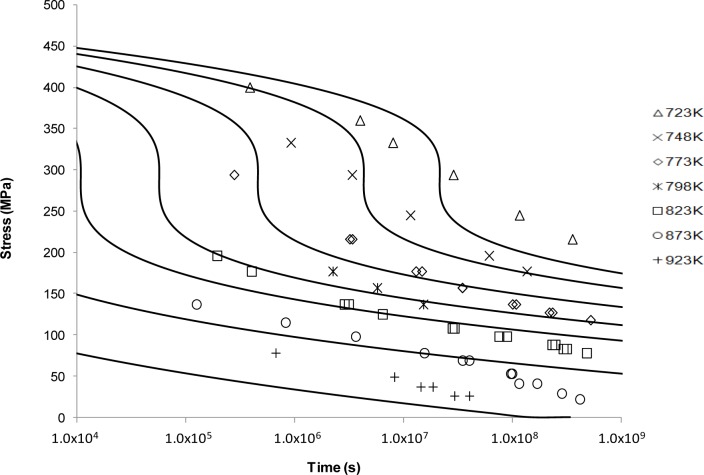
The Manson-Haferd’s stress *vs*. time to fracture predictive curves using Grade 22 Steel creep data.

Figure 3 shows the application of the Manson-Haferd approach. This fit shows a similar sigmoidal shape to Larson-Miller but with a substantially worse fit in this case. From Figure 3, it can be observed that the best fits are obtained at the intermediate temperatures. For the extreme temperatures, *i.e*., 723 and 923 K, the Manson-Haferd model fails to predict the experimental results. The exaggerated sigmoidal and flat shapes at 723 and 923 K, respectively, mean that the curves are unable to describe the actual creep properties at these conditions.

#### 4.3.3. The Orr-Sherby-Dorn Approach

**Figure 4.**
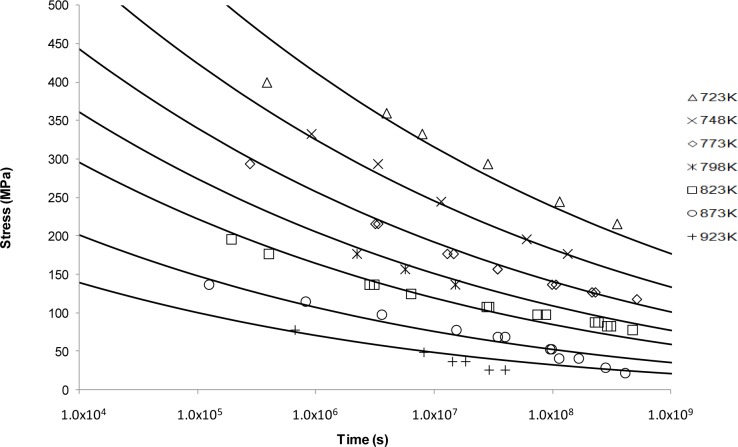
The Orr-Sherby-Dorn’s stress *vs*. time to fracture predictive curves using Grade 22 Steel creep data.

The OSD predictive curves are shown in Figure 4. This approach, unlike the previous ones, produced a smooth decaying curve. From these results it can be seen that this curve shape fits well with the longer life data at lower temperatures. It no longer provides a good fit for higher temperature data, nor the shorter rupture lives possibly due to changes in creep mechanism.

#### 4.3.4. The Manson-Succop Approach

**Figure 5.**
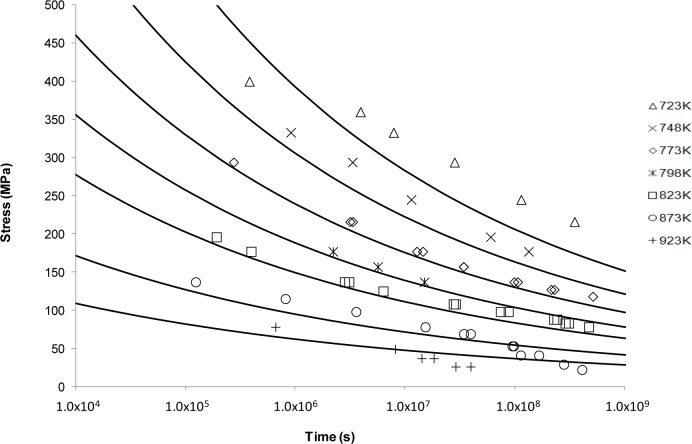
The Manson-Succop’s stress *vs*. time to fracture predictive curves using Grade 22 Steel creep data.

Figure 5 shows the results of applying the Manson-Succop approach to predicting creep rupture time. This method produced a similar creep curve shape to OSD shown in Figure 4. This is to be expected as these two methods are very similar with the difference between them lying in the time-temperature relationship (*T vs*. 1/*T*). However, this approach provides the best fit for creep rupture data at the intermediate temperatures and intermediate lifetimes.

#### 4.3.5. The Goldhoff-Sherby Approach

**Figure 6.**
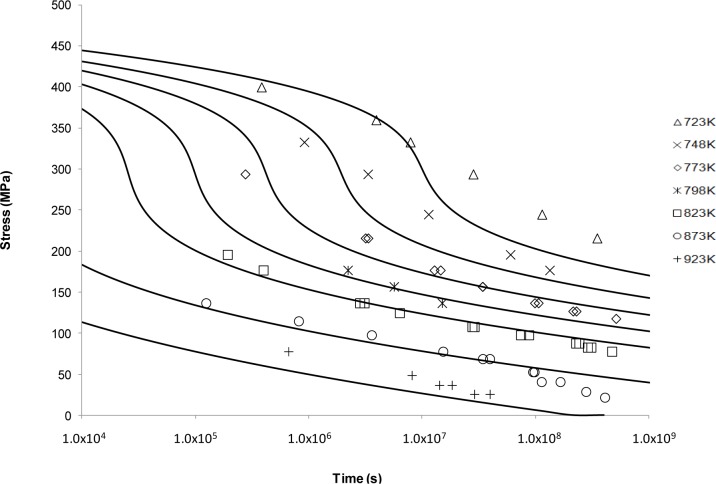
The Goldhoff-Sherby’s stress *vs*. time to fracture predictive curves using Grade 22 Steel creep data.

This approach, shown in Figure 6, produced similar results to the Manson-Haferd approach in Figure 3. The similarity of methods arises from their form with the difference arising from each approach’s time-temperature relationship (*T vs*. 1/*T*). Unlike the Manson-Haferd method, the Goldhoff-Sherby approach produces better fits to the experimental data with its best fits in the intermediate to high temperatures, long and very short lifetime range.

The above models are considered the most simplistic of those reviewed in this paper. It can be seen that they do not provide good fits to experimental data, and as such, are often implemented in a more flexible way. The more practical application of these models provides individual fits at each temperature such that their parameters function, *P*, change with temperature rather than being tied to an initially derived value [47–49]. This produces substantially better fits but also introduces the unknown dependency of these parameters, and hence constants, to temperature. It also means more tests need to be done in order to satisfy the minimum statistical threshold as each temperature is effectively a new “unique” set of data.

#### 4.3.6. The Soviet Prediction Approach

**Figure 7.**
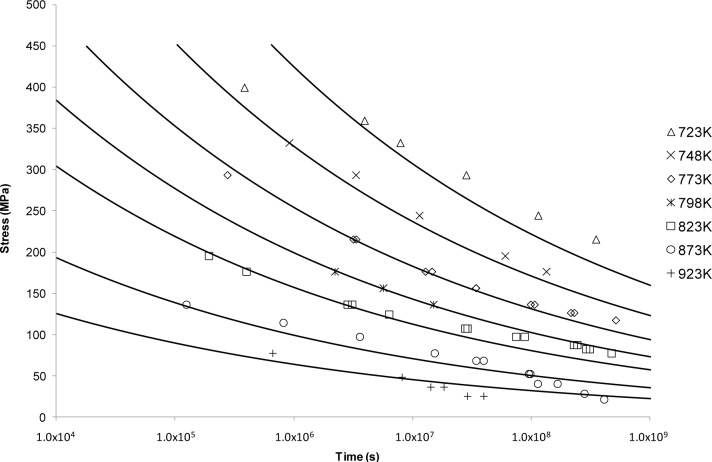
The stress *vs*. time to fracture curves of Soviet Model 1 using Grade 22 Steel creep data.

**Figure 8.**
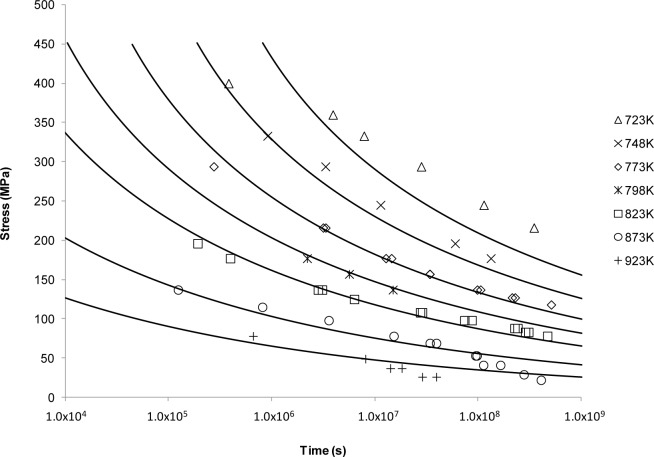
The stress *vs*. time to fracture curves of Soviet Model 2 using Grade 22 Steel creep data.

Figures 7 and 8 depict the predictive curves of the Soviet Prediction approach. Both Soviet 1 and Soviet 2 produced very flat σ *vs. t* curves that did not reflect the trend of the data. This is demonstrative of the point made by Evans [22] that extrapolation beyond the input data using these models was found to be highly inaccurate possibly due to the model not replicating the trending behaviour.

#### 4.3.7. The Minimum Commitment Approach

**Figure 9.**
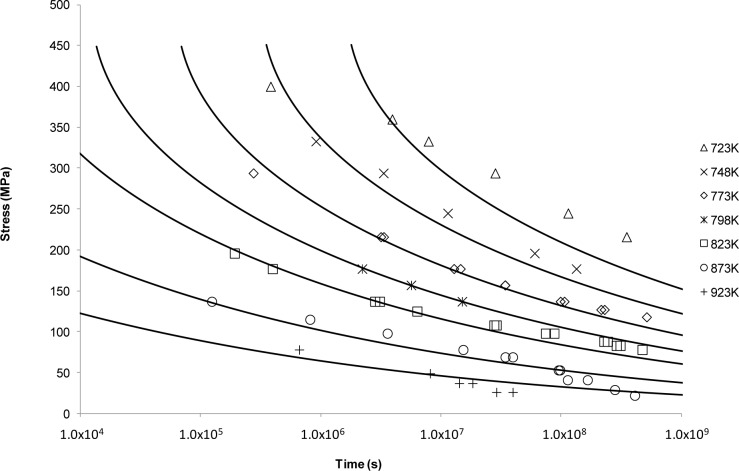
The stress *vs*. time to fracture curves of the Minimum Commitment method using Grade 22 Steel creep data.

Figure 9 displays the results obtained from the Minimum Commitment approach. This method is a vast improvement upon the previous ones as it provides a better fit to the experimental data and more importantly reflects the trend of the data. This is seen through the change in the curve shape from the highest to the lowest temperature. This shift in the curve shape and consequential good prediction may be attributed to the increased flexibility and sensitivity of the approach gained from having a larger number of constants.

#### 4.3.8. The Hyperbolic-Tangent Approach

**Figure 10.**
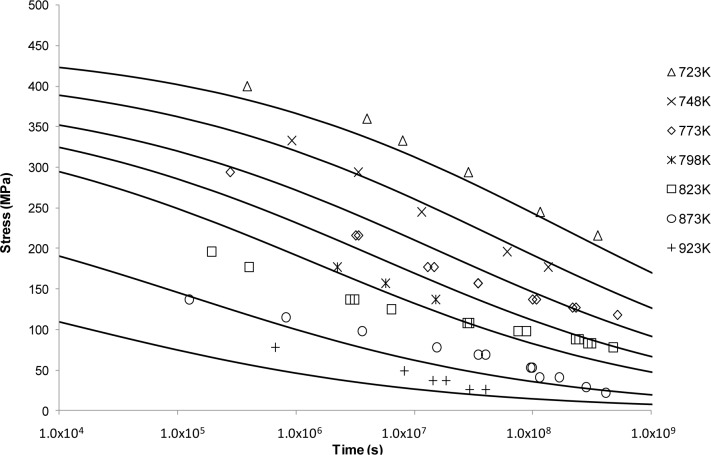
The Hyperbolic Tangent’s stress *vs*. time to fracture predictive curves using Grade 22 Steel creep data.

The alternative shape of stress-rupture curves produced by the hyperbolic tangent approach is evident in Figure 10. Using this approach, the constructed curves show reasonable fits to the observed creep behaviour as a result of the hyperbolic functions in its equation providing smooth curvatures. It is also observed that there is an inflection point at around 50% σ_TS_ at each corresponding temperature. This agrees with other observations [28–30] and implies that the creep mechanism is dependent on the applied stress level above and below σ_y_ (or σ_TS_). Another observation is that at intermediate temperatures (773, 823 and 873K) the curves deviate from the experimental data, in particular at the low stresses. This indicates that the shape of the outliers (highest and lowest temperature) dominate curve shapes and hence when using this method due care needs to be taken over the range of temperature it is applied to, and the amount of intermediate points included in the initial fitting of the constants.

#### 4.3.9. The Wilshire Approach

**Figure 11.**
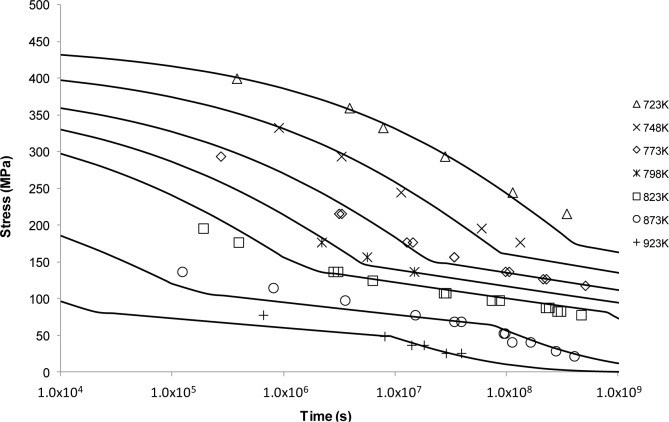
The Wilshire Equation’s stress *vs*. time to fracture predictive curves using Grade 22 Steel creep data.

Curves predicted by the Wilshire approach shown in Figure 11, are considered to be a good fit to the experimental measurements in both the high and the low stress regimes at all temperatures. It can also be observed from these curves that there are “kink” points at which the trend of the creep data changed according to the stress level involved (and hence a change in the constants *k*_1_ and *u*). In recent studies, it was found that these inflection points separate regions of different activation energies in different stress regimes [50] signifying the role deformation mechanisms have in determining creep behaviour.

#### 4.3.10. Errors and Accuracy

**Figure 12.**
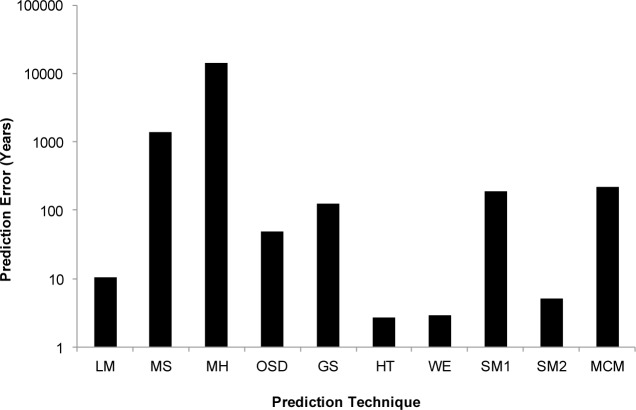
Error analysis of the conventional and modern predictive approaches.

The accuracy of each of the above approaches in predicting creep was evaluated using a Mean Squared Error (MSE) method where the error is calculated by:

$$\text{MSE}=\frac{1}{n}\sum_{t=1}^{n}{\left[t_{\text{actual}}-t_{\text{predicted}}\right]}^{2}$$ (26)

where *t*actual and *t*predicted are the experimental and predicted times to failure, respectively, and n is the number of the available data points. Error calculations shown in Figure 12 and in Table 1 were evaluated by taking the total number of error for all temperatures for each of the approaches. Whilst there are numerable approaches to error calculation, the MSE was considered the most applicable as it is an aggressive approach and it provides a simple and clear means to distinguish between the various approaches. In Figure 12 it can be seen that the simpler models return larger errors, whilst the more complex ones such as the SM2, Hyperbolic Tangent and Wilshire better represent the material’s creep properties.

Taking a closer look at Soviet Model 1 and Minimum Commitment approaches, these results should be expected. As highlighted when describing the approach, the Soviet and Minimum Commitment approaches rely on a large number of fitted constants meaning these equations have greater flexibility and sensitivity. Although this reproduces experimental data more accurately, it also means that these methods require greater care in application as the ability to produce unphysical curves or unreasonable constants is possible.

In considering the error associated with the two best methods, Hyperbolic Tangent and Wilshire, it is important to qualitatively and quantitatively consider each approach. The Hyperbolic Tangent approach provides a good fit to the creep-rupture data but from a visual inspection the region splitting Wilshire approach looks more accurate. The reason this is not reflected in the above error calculation is that the measurement of error is the difference in rupture times, which for the Wilshire approach produces an exaggerated error with higher temperatures due to the very flat nature of the curves produced. This limitation needs to be kept in mind when applying any creep lifing method as there are numerous ways to quantify error which may not reflect the quality of fit. In acknowledging these limitations, the Wilshire approach does not suffer the complexity of a large number of constants requiring the determination of only two constants which have so far have proven to be constant, although the constants vary in different regimes. Therefore, from this treatment it can be seen that the more complex methods provide better results when predicting creep properties with the most promising methods for now being the Wilshire and Hyperbolic Tangent approaches due to their relative simplicity and prediction capability.

### 4.4. Approaches to Reproducing Full-Creep Curves (Titanium IMI834 Data)

#### 4.4.1. The θ-Projection Approach

**Figure 13.**
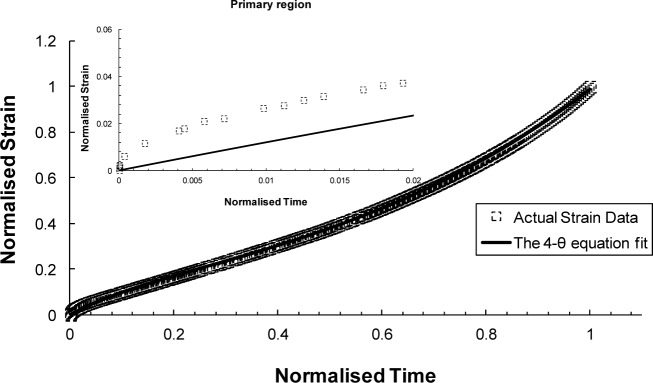
Creep curve for 823 K, 600 MPa fitted using the 4-θ approach.

**Figure 14.**
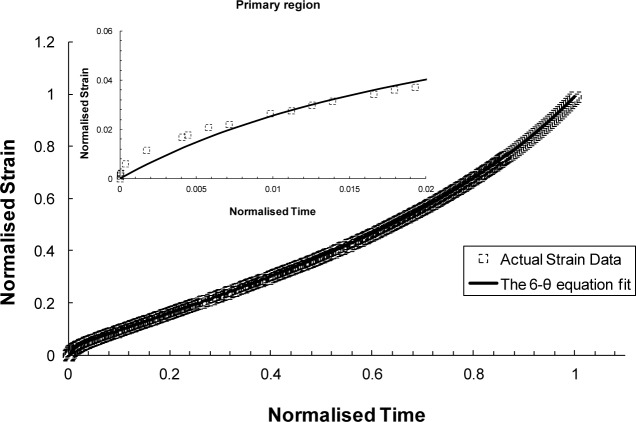
Creep curve for 823 K, 600 MPa fitted using the 6-θ approach.

The θ-projection approach was examined using the available Titanium IMI834 data. A comparison is shown in Figures 13 and 14 for the 4-θ and 6-θ methods, respectively. It is evident from these figures that the 6-θ method is more capable of describing the whole creep curve, in particular, providing a more accurate description of primary creep. The limitation of this approach is that full creep curves need to be available prior to carrying out analysis using this approach.

#### 4.4.2. The Uniaxial Creep Lifing (UCL) Approach

**Figure 15.**
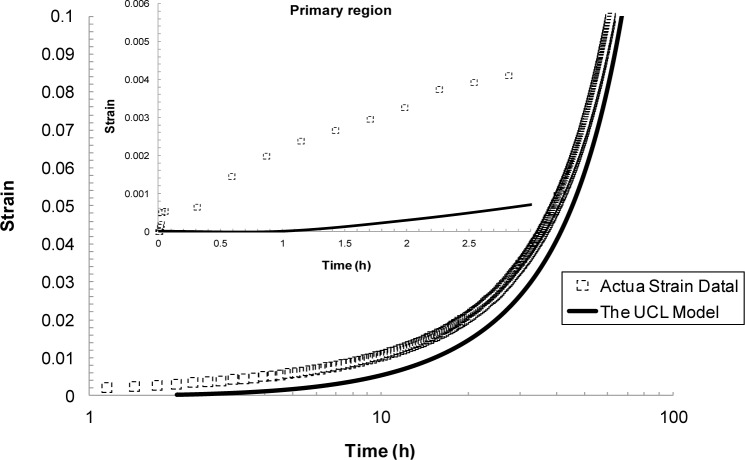
Creep curve for 923 K, 260 MPa predicted using the Uniaxial Creep Lifing approach.

The Uniaxial Creep Lifing approach is shown in Figure 15. From these results it can be seen that this approach was not able to accurately describe the primary creep region. This is not desirable for the aerospace industry as engineers are often faced with design problems that need to account for a material undergoing small strains.

#### 4.4.3. The Wilshire Approach

**Figure 16.**
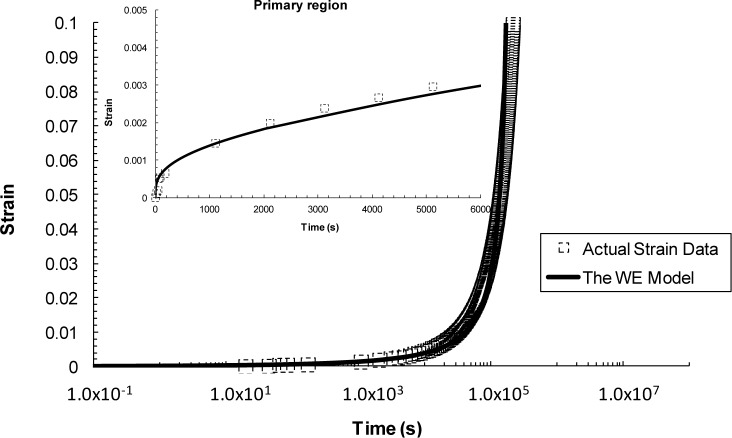
Creep curve for 923 K, 260 MPa predicted using the Wilshire approach.

The Wilshire technique has provided an impressive description of the whole creep curve, as well as, the primary region as seen in Figure 16. This description of the creep process stands alone as the most accurate description of the previously discussed methods.

Listed in Table 2 are the model equations used to fit the data for the creep rupture approaches used (Sections 4.3 and 4.4). In Table 3, the values of the constants for the various approaches are listed.

## 5. Discussion and Conclusions

The requirement for reliable methods of predicting long-term creep behaviour is more critical now than it has ever been. With conventional power plant building still widespread, the opportunity to increase operating temperatures and/or reduce material costs through higher stresses, is highly desirable but unrealised. One of the reasons for this is that there is little confidence in creep prediction on the timescales required for industrial applications without extensive and protracted testing.

The limitations of traditional power law based approaches have long been recognised [51] although designers have been reticent to move away from these approaches due to their simplicity and ease of use. Presumably, this is also because no alternative approach offers a significant enough increase in confidence to warrant a change in creep behaviour prediction and characterisation methodology. The Larson-Miller approach shown in Figure 2 aptly demonstrates the nature of the problem. Fits based on rupture data seem reasonable for lower temperatures and the behaviour of the material is well described. However, for higher temperatures the accuracy of the fits decreases markedly and non-conservative predictions of rupture time occur. Obviously this presents a significant problem and results in the undesired need for further testing.

Looking at the next generation of creep lifing approaches provides no better alternative to the Larson-Miller approach. This is evident from the Manson-Haferd approach providing poor fits at the highest and lowest temperatures, the Orr-Sherby-Dorn approach fitting poorly at short lifetimes and high temperatures, the Manson-Succop approach fitting poorly at the temperature extremes and short lifetimes, and the Goldhoff-Sherby approach fitting poorly at low temperature and short lifetime. To try and overcome this, more complex models were developed. The Soviet Prediction approach offers better fits, but has very limited extrapolation ability and inherits complexity due to the number of constants that need to be fitted. The Minimum Commitment approach offers good fits but still suffers the need to fit a large number of constants to sample data.

Regaining simplicity in implementation by reduction of constants, both the Hyperbolic Tangent and Wilshire approaches provide good fits with minimum associated complexity. For the Hyperbolic Tangent approach developed by Rolls-Royce plc, the creep curves trend to σ_TS_ for each temperature as *t* → 0, and trend to an infinite life as σ → 0. This represents the expected physical behaviour of the material under such conditions. As such, the Hyperbolic Tangent approach has been applied to a number of materials [30] and clearly offers a step forward in prediction of creep. However, the complex behaviour of Grade 22 steel provides problems which the technique is unable to accommodate, seen by poor prediction of long rupture times between 773 and 873 K. Despite the fact that the Hyperbolic Tangent approach produces the lowest MSE in comparison to the Wilshire approach, it is clear that the changing trends in the creep-rupture data are not always accommodated. The issue here is that the Hyperbolic Tangent approach offers a point of inflection at σ = 0.5σ_TS_, which presumably is not offering enough flexibility in the curve shape to accommodate the behavior of the material in question.

The Wilshire equations share a number of common features with the Hyperbolic Tangent approach including providing good fits to experimental creep data. One of the common features between approaches is stress normalisation and the creep curve trending towards infinite life as σ→0. Key publications have sought to rationalise the assumptions made when using the equations, and in particular the behaviour of materials considered [8,16,31,32]. Whilst normalisation by σ_TS_ is particularly useful in reducing batch to batch scatter, perhaps the most important element in applying the equations is the ability of this approach to “react” to creep mechanism regimes as shown in Figure 11. It is apparent that the first “transition” (change in *k*_1_ and *u*), occurs at the yield stress of the material. This change in behaviour is perhaps unsurprising due to changes in dislocation behaviour which occur above and below the yield stress. Below the yield stress creep occurs through the movement of pre-existing dislocations and mainly by grain boundary zone deformation. Above the yield stress, new dislocations are continuously generated which is often noticeable by a more extended primary phase that is limited only by exhaustion when strain hardening offsets dislocation generation. As this decaying process is offset by an accelerating tertiary phase, which is brought about by damage such as cavitation and triple point cracking, a minimum rather than secondary creep rate occurs. Previous studies have inferred these mechanisms from changes in elongation and reduction in area measurements of creep tests above and below yield [52]. The second transition occurs at high temperature, *i.e*., 798–923 K, and relatively long rupture times is due to degradation of the material through overaging. The bainitic structure evident prior to testing begins to break down and the microstructure evolves to ferrite with coarse molybdenum carbide particles. Interestingly, activation energies calculated using the Wilshire approach well represents these changes in behaviour. Above the yield stress, creep occurs within the grains and the activation energy calculated for creep is 280 kJ·mol^−1^, approximately the value for self diffusion in the ferrite lattice. Below the yield stress, the value drops to 230 kJ·mol^−1^ as deformation is confined to the grain boundary zones. However, the activation energy for low stresses/high temperatures returns to 280 kJ·mol^−1^, as following the degradation of bainite to ferrite, creep returns to grain interiors in this effectively weaker material [8].

When considering the MSE error analysis for predicted creep life alongside the criteria provided by the ECCC, it is clear from both Figure 12 and Table 1, that the Wilshire and hyperbolic Tangent approaches provide the most accurate fits overall with the Wilshire Equations being most accurate for a number of temperatures. Interestingly, unlike the power law methods, the curve shape produced by the Wilshire is not its most significant advantage. Rather, the ability of the approach to react to creep mechanism changes which have been observed, offers a greater advantage as the underlying physical phenomena are represented within the approach. The effectiveness of this technique in predicting 100,000 h creep life based on only 5000 h has been demonstrated for a number of alloys [8,53]. It should also be noted here that the “region splitting” technique (transition) utilised in the Wilshire approach is not a wholly original approach with previous examples, particularly by Kimura *et al.* [54], worth consideration.

In summarizing the full creep curve approaches, we can see a greater emphasis on representing the underlying physical processes of creep. The most established of these methods is θ-Projection which relies on extrapolating from known curves to unknown curves with dependence on the same physical phenomena being present in both cases. The additional term in the 6-θ approach provided a better description of the primary creep than compared to the 4-θ. Taking a more fundamental route, the Uniaxial Creep Lifing approach is mechanistically-based and represents a group of models that try to encompass the underlying physical process of creep. In literature, replication of creep curves via this approach has been reasonable even though in this review the method poorly predicted the primary phase of the creep curve [39]. Wilshire offers an additional approach that lies between the θ-Projection and the Uniaxial Creep Lifing methods. The Wilshire approach, as previously discussed, is a model that is sympathetic to the underlying creep mechanism without explicitly defining it. As such, it is a combination of a physically independent and a mechanistically-defined model which produces good, if not better, fits than the other approaches reviewed here.

In considering the requirements of the aerospace and power generation industries, the approaches reviewed here provide a summary of the past and an insight into the future. Implementing power law-based creep methods in the modern world is becoming harder to justify with greater computing power available to deal with more complex accurate models such as the Hyperbolic-Tangent, Soviet, Minimum Commitment and the Wilshire approaches. For the whole creep curve prediction, it can be seen that incorporating the underlying physical phenomena is producing more accurate models that encapsulate and enhance our understanding of the processes materials experience. Clearly, collaboration within the community would offer opportunities for further development of these approaches and as such bring closer to realisation the goal of a comprehensive creep behavior model.

## Figures and Tables

**Table 1.** Error values for the conventional and modern predictive approaches.

Temperature (K)	Errors in each extrapolation technique (Years)										Minimum Error
	LM	MS	MH	OSD	GS	HT	SM1	SM2	MCM	WE	
723	2.9406	3.783	3.819	1.824	4.03	1.541	3.0745	3.674	3.6367	2.425	HT
748	0.657	1.243	0.67	0.095	1.007	0.582	0.8212	1.022	1.05086	1.072	OSD
773	1.5671	3.171	356.3	1.489	25.46	2.038	2.7496	2.178	2.60138	0.376	WE
798	0.1366	0.079	0.523	0.109	0.397	0.408	0.0051	0.035	0.02743	0.056	SM1
823	1.0737	4.125	532.8	3.822	32.44	4.249	4.2348	3.284	3.63002	5.621	LM
873	22.675	3006.4	30,968	108.1	274.2	2.819	407.24	1 × 10^−7^	477.314	0.641	SM2
923	0.4621	36.804	0.613	5.303	0.382	0.515	12.078	13.91	11.2147	0.145	WE

**Table 2.** Equations and parameters of creep life approaches.

Creep Lifing Technique	Stress Function	Note
LM	P_LM_ = −1.44 × 10^−6^σ^4^ + 8.048 × 10^−4^σ^3^ + 2.73 × 10^−2^σ^2^ − 9.21×10σ + 57175.13	constants explained in Table 3
MH	P_MH_ = 3.54×10^−11^σ^4^ − 7.83 × 10^−8^σ^3^ + 5.07 × 10^−5^σ^2^ − 1.311 × 10^−2^σ + 1.464	constants explained in Table 3
OSD	σ = 4 × 10^−7^ [(44250/T) − log*t*]^5.3787^	constants explained in Table 3
MS	σ = 4 × 10^19^ [log*t* + 0.0705T]^−9.389^	constants explained in Table 3
GS	PGS = −9.63 × 10^−6^σ^4^ − 5.51×10^−3^σ^3^ + 1.017 × 10σ^2^ − 3.65 × 10^3^σ + 5.609	constants explained in Table 3
HT	σ = UTS/2 (1 − tanh[*k*log(*t*/*t*_i_)])	*k* is constant
WE	σ/UTS = exp (−k_1_[t_f_·exp(−*Q*_c_*/*RT*)]*^u^*)	*u* and *k*_1_ = *f*(σ*)*
SM1	log*t* = *a* + *b*log*T* + *c*logσ + *d*/*T* + *f*·*σ/T*	constants explained in Table 3
SM2	log*t* = *a* + *b*log*T* + *c*(logσ)/*T* + *d*·*σ/T* + *f/T*	constants explained in Table 3
MCM	log*t* = *a* + *b*logσ + *c*·σ + *d*·σ^2^ + *fT* + *g/T*	constants explained in Table 3

**Table 3.** Determined constants of creep life approaches.

Category	Method	Parameters	Note
Creep Lifing Techniques	LM	*C*_LM_ = 41.5	constant
	MH	log*t*_a_ = −77.6 *T*_a_ = 986	constant
	OSD	*C*_OSD_ = 44250	constant
	MS	*C*_MS_ = 0.0705	constant
	GS	log*t*_a_ = −48	constant
		1/*T*_a_ = 0.00093	
	HT	*k* = 0.144, *t*_i_ = exp(−0.007*T* + 7.7992)	*t*_i_ = *f*(*T*)
	SM1	*a* = 410.05, *b* = −53.734, *c* = −6.99, *d* = 4.68, *f* = −0.24	constants
	SM2	*a* = 560.26, *b* = −77.68, *c* = −7129.34, *d* = 8.3, *f* = 14363.1	constants
	MCM	*a* = 101.83, *b* = −6.47, *c* = −0.0139, *d* = 0.000027, *f* = −0.065, *g* = 2.0097	constants
	WE	Low stress regime: *u* = 0.2944, *k*_1_ = 624 Intermediate stress regime: *u* = 0.0714, *k*_1_ = 6.45 High stress regime: *u* = 0.3072, *k*_1_= 3584	*u* and *k*_1_ = *f*(σ)
Full creep representation (923 K/260 MPa)	θ-projection	4-θ method: θ_1_ = 0.9244, θ_2_ = 0.3118, θ_3_ = 0.0162, θ_4_ = 3.833 6-θ method: θ1 = 30.00021, θ_2_ = 0.00966, θ_3_= 0.01297, θ4 = 3.99825, θ_5_ = 0.00064, θ_6_ = 1000.284	θ = *f* (σ*, T*)
	WE	Low stress regime: *w* = 0.21, *k_3_* = (3.815484/ε^0.8^) + 350 High stress regime: *w* = 0.8, *k_3_* = (3 × 10/ε) + 2 × 10	*w* = constant, *k* = *f* (ε)
	UCL	*k* = 0.000695, *t*_tr_ = 3.34069, *M′* = 31.092	*k*, *t_tr_*, and *M*′ = *f*(σ)
